# Ajmaline-induced Brugada Phenocopy, Right Bundle Branch Block, or Both?

**DOI:** 10.19102/icrm.2021.120906

**Published:** 2021-09-15

**Authors:** Habibe Kafes, Irem Dilara Can, Nezaket Merve Yaman, Meryem Kara, Ahmet Korkmaz, Ozcan Ozeke, Serkan Cay, Firat Ozcan, Serkan Topaloglu, Dursun Aras

**Affiliations:** ^1^Department of Cardiology, University of Health Sciences, Ankara City Hospital, Ankara, Turkey

**Keywords:** Ajmaline, Brugada phenocopy, Brugada syndrome, right bundle branch block

## Abstract

In equivocal or suspected cases of Brugada syndrome (BrS), ajmaline testing is frequently used in the diagnostic approach. However, the administration of sodium channel blockers can not only elicit the coved ST-segment elevation characteristic of type 1 Brugada pattern but also induce right bundle branch block (RBBB), which can preclude the electrocardiographic manifestations of BrS. We describe a case report wherein RBBB posed a diagnostic challenge during the ajmaline test for suspected BrS.

## Case presentation

**Figure 1:**
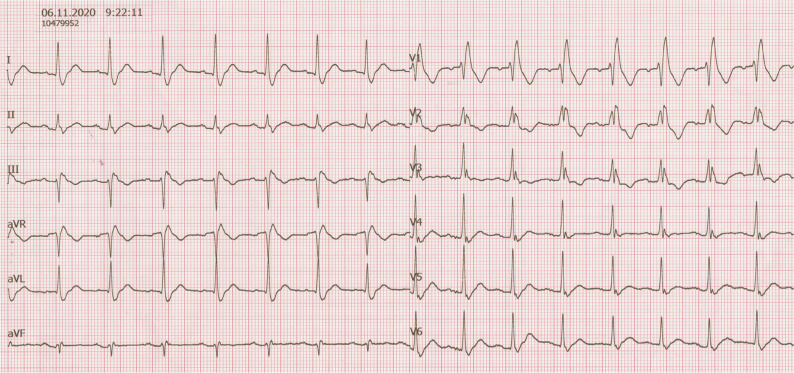
Initial 12-lead ECG recorded at the emergency department, showing sinus rhythm with RBBB.

A 28-year-old Caucasian man was referred to our center because of a possible right bundle branch block (RBBB) electrocardiographic (ECG) pattern recorded during a medical evaluation for suspected coronavirus disease 2019 infection **(Figure 1)**. His physical examination was unremarkable. There was no family history of syncope or sudden cardiac death.

**Figure 2:**
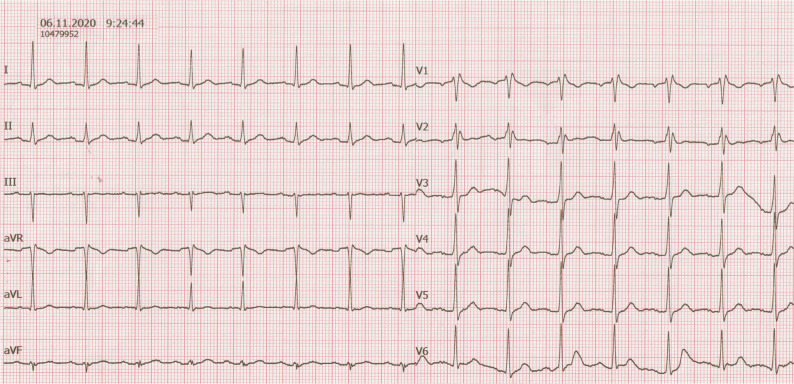
A higher intercostal space ECG, showing the resolution of the RBBB.

**Figure 3:**
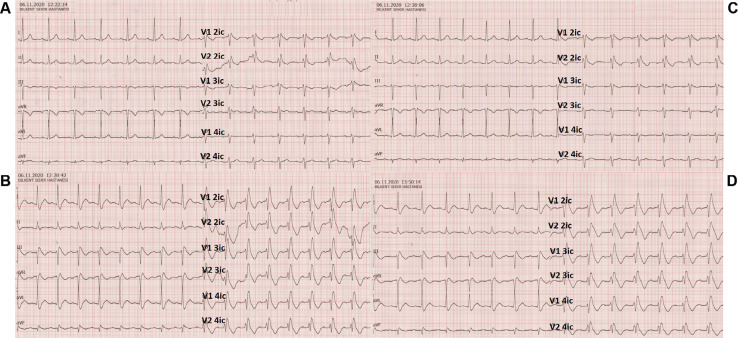
The ajmaline-challenge test, performed twice to confirm the reproducibility of findings in specifically designed electrograms covering the right ventricular outflow tract, showing a type 1 BrS–like ECG pattern superimposed on the pre-existing atypical RBBB.

At first glance, the patient had sinus rhythms with an atypical appearance of RBBB. Indeed, RBBB is generally considered a benign finding that does not imply an increased risk when found in asymptomatic healthy individuals.^1^ However, the presence of it in subjects affected by cardiomyopathies or channelopathies may conceal the underlying ECG changes, thus making the diagnosis of such conditions more challenging. Its atypical appearance in the current case led us to consider that a type 1 Brugada ECG pattern would be hidden or “masked” by the underlying RBBB^2^; then, we ordered a higher intercostal space ECG, which showed the resolution of RBBB **(Figure 2)**. However, although the changes related to the RBBB also resolved on the frontal leads, we could not rule out a history of intermittent RBBB. The diagnostic drug challenge performed by intravenous administration of ajmaline (1 mg/kg over five minutes) by specifically designed electrograms covering the right ventricular outflow tract^3^ **(Figure 3A)** unmasked a diffuse abnormal response compatible with the type 1 Brugada syndrome (BrS)-like ECG pattern superimposed on the pre-existing atypical RBBB during 30 seconds of infusion **(Figure 3B)**, and the infusion was immediately stopped at that stage. After complete resolution of the electrogram **(Figure 3C)**, we readministered ajmaline, which also showed ST-segment elevation in the precordial leads as a reproducible finding **(Figure 3D)**.

## Discussion

Indeed, Brugada and Brugada initially described an RBBB pattern among their eight cases of BrS.^3^ Thereafter, it was shown that, although the presence of the RBBB pattern is not necessary for reaching a diagnosis of BrS, it is supportive. In RBBB in healthy subjects, the ST segment is usually not elevated in the right precordial leads. In RBBB patients, activation of the entire right ventricle was delayed, while, in contrast, delayed activation was confined to the right ventricular outflow tract in BrS patients.^4^ Therefore, BrS patients do not have the wide final S-waves in the left leads (I, aVL, V5, and V6) necessary for RBBB. However, the presence of a wide and/or large S-wave in lead I has been found to be a powerful predictor of life-threatening ventricular arrhythmias in patients with BrS.^5^ Furthermore, the characteristic ECG pattern of BrS can be masked by complete RBBB and exposed by the resolution of the block or pharmacological or pacing maneuvers.^2,6,7^ In the presence of complete RBBB, the premature activation of the right ventricle by apical pacing caused fusion beats, with narrower QRS complexes disclosing the distinctive coved-type ST-segment elevation in the right precordial leads.^8^ Thus, BrS should be ruled out whenever a slight ST-segment elevation is observed in the right precordial leads in the presence of RBBB.^6,8^

In equivocal or suspected cases of BrS, ajmaline testing is frequently used in the diagnostic approach.^9–12^ However, some authors have reported a potential 5% false-positive rate, thus making the universal interpretation of provocative drug testing challenging.^13,14^ Therefore, not only diagnostic decisions but also therapeutic choices with long-term implications were adopted worldwide before the potential for false-positive results of the ajmaline test was fully appreciated.^13^ Clinicians should consider all alternative causes in unexplained cardiac arrest and avoid ajmaline doses of greater than 1 mg/kg.^15^ Furthermore, it should be kept in mind that the administration of sodium channel blockers could not only elicit the coved ST-segment elevation characteristic of type 1 Brugada pattern but also induce an RBBB that can preclude the ECG manifestations of BrS.^16^ As placing the right precordial ECG leads at an upper position (second or third intercostal space) increases the sensitivity of ECG to detect all types of BrS,^3^ it would be nice to take an ECG at upper intercostal spaces and perform an ajmaline test to unmask the true RBBB effect.
